# Bioactive compounds as therapeutic modulators of metabolic syndrome: targeting inflammation and gut microbiota regulation

**DOI:** 10.3389/fphys.2026.1766078

**Published:** 2026-04-28

**Authors:** Magdalene Eno Udobi, Mercy Bella-Omunagbe, Israel Sunmola Afolabi, Shalom Nwodo Chinedu

**Affiliations:** 1 Department of Biochemistry, College of Science and Technology, Covenant University, Canaanland, Ota, Ogun State, Nigeria; 2 Covenant Applied Informatics and Communication Africa Centre of Excellence (CApIC-ACE), Covenant University, Canaanland, Ota, Ogun State, Nigeria; 3 Covenant University Public Health and Wellbeing Research Cluster (CUPHWERC), Covenant University, Ota, Ogun State, Nigeria

**Keywords:** food bioactives, gut microbiota, inflammation, insulin resistance, metabolic syndrome

## Abstract

Food bioactives, including polyphenols, flavonoids, omega-3 fatty acids, and glucosinolates, play a crucial role in preventing metabolic syndrome by modulating chronic inflammation, gut microbiota homeostasis, and metabolic processes. These compounds influence key molecular pathways implicated in metabolic dysfunction and systemic inflammation. This review explores the mechanisms through which food bioactives contribute to metabolic health, emphasizing their role in inflammation regulation, gut microbiota modulation, and insulin sensitivity. A comprehensive literature review was conducted using databases such as PubMed, Scopus, and Web of Science. Relevant peer-reviewed articles, meta-analyses, and clinical trials published in the last two decades were analyzed, focusing on bioactives’ biochemical actions and therapeutic potential in Metabolic syndrome. The study showed that bioactives mitigate inflammation by inhibiting NF-κB signaling and NLRP3 inflammasome activation, reducing pro-inflammatory cytokines (TNF-α, IL-6, IL-1β). They also modulate gut microbiota, promoting beneficial bacteria (e.g., *Akkermansia muciniphila*) and enhancing gut barrier integrity via increased expression of tight junction proteins. Short-chain fatty acids (SCFAs) derived from microbial metabolism contribute to systemic anti-inflammatory effects. Clinical studies indicate that polyphenol-rich diets, such as the Mediterranean diet, improve metabolic syndrome parameters by lowering inflammatory markers, enhancing lipid profiles, and improving insulin sensitivity. Despite promising findings, challenges such as poor bioavailability and variability in gut microbiome responses hinder clinical application. Strategies like nanoencapsulation and microbiome-targeted nutrition may optimize bioactive efficacy. Overall, food bioactives represent a promising strategy for metabolic health. Future research should focus on enhancing bioavailability, personalized nutrition, and large-scale clinical trials to establish optimal dosing and long-term benefits.

## Graphical Abstract

**Figure:**
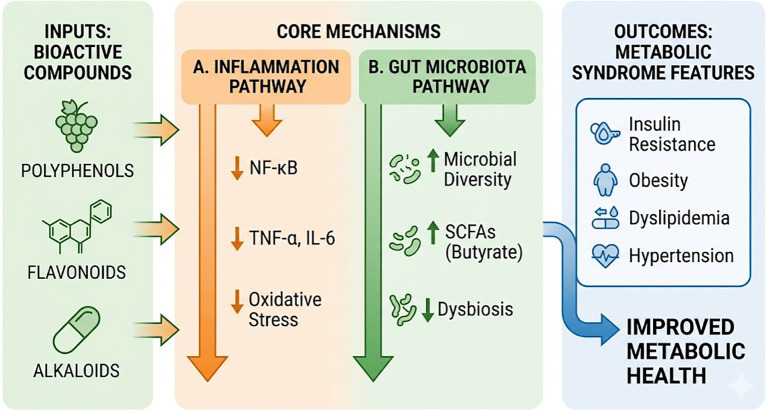
Various sources of food bioactives (Source: Effiong et al., 2025a).

## 1 Introduction

Bioactive compounds in food refers to substances that confer additional benefits beyond their nutritional value. They are also referred to as functional foods (Ma et al., 2025; Mafe et al., 2025). These substances include a wide range of compounds such as polyphenols flavonoids, omega-3 fatty acids, fiber, prebiotics, probiotics, and many other phytochemicals (Dama et al., 2024; Xavier et al., 2024). They are found in fruits, whole grains, vegetables and other food sources. They stand out as functional foods because of their anti-inflammatory, immunomodulatory, and antioxidant properties (Shahidi and Danielski, 2024; Ma et al., 2025). Flavonoids are a class of bioactive compounds that belong to a subclass of polyphenols, their major sources include citrus fruits, tea, berries, etc (Effiong et al., 2024a; Effiong et al., 2025a). They function in reducing oxidative stress and modulating cellular signal transduction pathways through their antioxidant capacities and anti-inflammatory properties (Effiong et al., 2025b). Omega-3 fatty acids are also a very vital class of food bioactives that are sourced from fish oils and function in preventing inflammation and cardio-related diseases (Das et al., 2024; Patted et al., 2024). Prebiotics and probiotics refer to a group of bioactive compound that possess the ability to alter the microbiome composition, preventing its dysbiosis and while improving the intestinal health and immune response of the body (Latif et al., 2023; Al-Habsi et al., 2024; Victoria Obayomi et al., 2024). Dietary fiber, a food bioactive, possess vasts functions. They act as substrates to the gut microbes, enabling them to produce short chain fatty acids (SCFA) which improves the regulation of inflammation and boosts immune response, glucose metabolism and lipid profiles (Fu et al., 2025). They also help in eliminating wastes products from the colon by increasing stool weight for easy excretion (Fu et al., 2022).

Metabolic syndrome can be referred to as a group of related diseases associated with the imbalance of the body’s homeostasis such as glucose, blood pressure, mineral, acid-base imbalances (Martemucci et al., 2023; Mohamed et al., 2023). These group of diseases include obesity, hypertension, dyslipidemia, diabetes, cardiovascular diseases, and other chronic pathological conditions. Major hallmarks of metabolic syndrome include lipid dysregulation, insulin resistance, inflammation, gut microbiota dysregulation, hypertension, genetic and epigenetic alterations, etc (Jha et al., 2023; Zhao et al., 2023). Overall, Chronic low-grade inflammation and gut dysbiosis play pivotal roles in the pathogenesis of metabolic syndrome. Inflammation is a natural response of the body to triggers in the immune system such as injury, infection or a deleterious stimuli, with the aim of conferring protection from damage or eliminating damaged cells and initiating return to normalcy (Scheithauer et al., 2020; Zhao et al., 2023; Acevedo-Román et al., 2024; Secchiero et al., 2024). This immune response is accompanied by the release of pro-inflammatory cytokines such as tumour necrosis factor- alpha (TNF-α) and interleukins (IL-6, IL-1, IL-8, IL-12) by the immune cells (Acevedo-Román et al., 2024; Bhol et al., 2024). Despite the beneficial nature of this immune response through the release of pro-inflammatory actors, a prolonged or excessive cytokine release can lead to chronic inflammation which then becomes deleterious rather than beneficial (Bhol et al., 2024). This can then further lead to a disruption in key signal transduction pathways, in this case, disruption of insulin signalling, thereby contributing to insulin resistance.

The gut is made up of diverse micro-organisms which made up its community. These microbes are crucial to the proper functioning of the gut, they regulate inflammation, immune response and produce metabolites that function in signal transduction, among diverse other functions (Hou et al., 2022). As a result, an imbalance in the gut microbial community, commonly referred to as a state of gut dysbiosis contributes to metabolic dysfunction by promoting endotoxemia and systemic inflammation (Menafra et al., 2024; Trehan et al., 2024).

Dietary interventions are crucial in modulating metabolic pathways and mitigating the risks associated with metabolic syndrome (Scaglione et al., 2025). Consuming bioactive-rich foods can influence key mechanisms underlying Metabolic syndrome, including inflammation, oxidative stress, and gut microbiota composition (Rajewski et al., 2025). Polyphenols, a class of bioactive compounds have been found to reduce oxidative stress by scavenging free radicals and upregulating antioxidant defense systems (Abdulghani and Al-Fayyadh, 2024). Omega-3 fatty acids can modulate lipid metabolism, reduce triglyceride levels, and attenuate inflammatory responses. Likewise, prebiotics and probiotics foster gut microbial diversity, leading to improved metabolic and immune homeostasis (Sullivan and Jones, 2024). Furthermore, dietary fiber enhances satiety, reduces postprandial glucose spikes, and lowers cholesterol levels (Alahmari, 2024). These enormous roles of food bioactive compounds in modulating metabolic syndrome and its associated hallmarks, makes them crucial to be studied.

Although, the link between diet and metabolism is well-established, a comprehensive synthesis of how specific molecular pathways intersect with microbial shifts remains fragmented. Therefore, this review aims to bridge this gap by critically evaluating the mechanisms through which food bioactives contribute to metabolic health. Distinctively, it focuses on the synergistic interplay between inflammation regulation, gut microbiota modulation, and insulin sensitivity. Furthermore, the review provides a unique analysis of the barriers to clinical efficacy, specifically addressing the complexities of bioavailability and inter-individual variability that often lead to inconsistent therapeutic outcomes.

## 2 Method

A structured methodology was employed to evaluate the therapeutic potential of bioactive compounds in managing Metabolic Syndrome (MetS), specifically focusing on their ability to regulate gut microbiota and suppress systemic inflammation. A systematic search was conducted across the PubMed, Scopus, and Web of Science databases for peer-reviewed literature, utilizing targeted Boolean strings that intersect metabolic markers (e.g., insulin resistance, dyslipidemia) with molecular mechanisms (e.g., NLRP3 inflammasome, short-chain fatty acids). Duplicates were removed and the study synthesized evidence from *in vitro*, *in vivo*, and clinical models. The final analysis categorizes the biological outcomes into four primary pillars: food bioactives, gut microbiome regulation, inflammation and metabolic syndrome.

## 3 Metabolic syndrome

Metabolic syndrome is a group of interrelated conditions that increase the risk of cardiovascular disease, type 2 diabetes, and other metabolic abnormalities. This syndrome is characterized by central obesity, insulin resistance, hypertension, hyperlipidemia, and chronic low-grade inflammation (Mafe et al., 2025). The complex biological mechanisms underlying metabolic syndrome involve genetic, environmental, and lifestyle factors that disrupt metabolic homeostasis and lead to systemic dysfunction (Thomaz et al., 2024).

Insulin resistance is a major central hallmark of metabolic syndrome, where tissues such as muscle, liver, and adipose become less responsive to insulin signaling. This impaired insulin sensitivity leads to hyperglycemia and compensatory hyperinsulinemia (Apostolopoulou et al., 2025). Over time, chronic insulin resistance results in beta-cell dysfunction and the development of type 2 diabetes. The molecular basis of insulin resistance involves defects in insulin receptor signaling, increased free fatty acid (FFA) levels, and chronic inflammation (Chandrasekaran et al., 2024). Elevated FFAs interfere with insulin signaling pathways and promote lipid accumulation, leading to ectopic fat deposition in non-adipose tissues such as the liver and skeletal muscle (Elkanawati et al., 2024).

In metabolic syndrome, adipose tissue has a key role in the disease process. Obesity leads to the growth in size and number of adipose tissue cells, which causes these cells to malfunction (De Sanctis et al., 2025). The malfunctioning cells release more pro-inflammatory cytokines like TNF-α, IL-6, and resistin, and less anti-inflammatory adipokines like adiponectin. This imbalance contributes to widespread inflammation, resistance to insulin, and problems with blood vessels (Uti et al., 2025). Additionally, the breakdown of fat in adipose tissue releases free fatty acids into the bloodstream, worsening metabolic issues (Kirichenko et al., 2022).

## 4 Hallmarks of metabolic syndrome

**Figure 1:**
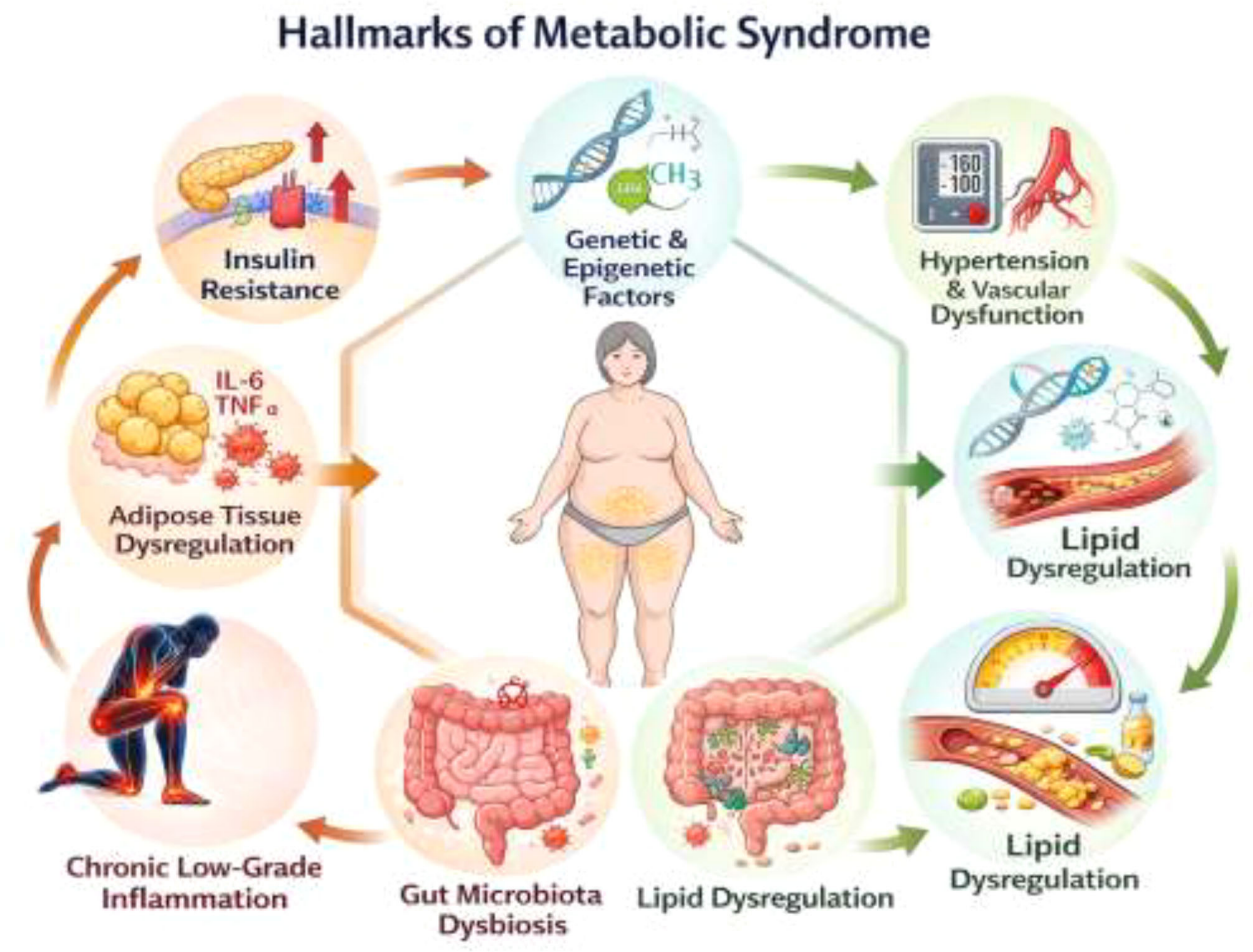
Hallmarks of metabolic syndrome.

The complex biological mechanisms underlying metabolic syndrome involve genetic, environmental, and lifestyle factors that disrupt metabolic homeostasis and lead to systemic dysfunction (Islam et al., 2024). Additionally, it involves various hallmarks such as insulin resistance, adipose tissue dysfunction, chronic inflammation, gut microbiota, lipid dysregulation, and genetic predispositions (**Figure 1**).

### 4.1 Insulin resistance

Insulin resistance refers to a condition characterized by the inability of the body to respond adequately to insulin, a hormone that functions in regulating the blood sugar by converting excess glucose to glycogen, especially in the muscles, liver and fat cells (Ahmed et al., 2021). In the presence of insulin resistance, these cells do not respond to the insulin hormone, resulting in excess blood sugar (hyperglycemia) (Elkanawati et al., 2024). As a feedback mechanism, the pancreas, produces more insulin (hyperinsulinemia) to aid reduce the high glucose levels. Overtime, the insulin-producing beta cells in the pancreas experiences a wear out, leading to type 2 diabetes and a dysregulation in insulin signal transduction (Rachdaoui, 2020). The development of insulin resistance characterized by high insulin and glucose levels, results in an alternative glucose storage as free fatty acids (FFA) which are then stored in key organs like the liver, muscles and around the stomach areas. The accumulation of fat cells increase the release of inflammatory cytokines which further disrupts insulin signaling (Jin et al., 2023).

### 4.2 Adipose tissue dysfunction

Adipose tissue plays a central role in the pathophysiology of metabolic syndrome. In obese individuals, adipose tissue undergoes hypertrophy and hyperplasia, resulting in adipocyte dysfunction (Velastegui et al., 2025). Dysfunctional adipocytes secrete increased levels of pro-inflammatory cytokines such as tumor necrosis factor-alpha (TNF-α), interleukin-6 (IL-6), and resistin while reducing the production of anti-inflammatory adipokines like adiponectin (Uti et al., 2025). This dysregulation contributes to systemic inflammation, insulin resistance, and vascular dysfunction. In addition, the increased lipolysis in adipose tissue releases FFAs into circulation, exacerbating metabolic disturbances (Lee et al., 2023).

### 4.3 Chronic low-grade inflammation

Chronic low-grade inflammation is a hallmark of metabolic syndrome and is driven by both innate and adaptive immune responses. It is a hidden but harmful type of inflammation associated with people with high blood sugar, belly fat and high blood pressure (Alemany, 2024). It differs from acute inflammation, in that is occurs for a prolonged period of time and is often caused by overactivity of the immune system (Alemany, 2024). The presence of fat tissues in the liver and other parts of the body trigger certain inflammatory pathways such as NFkB and Nodes-Like Receptor Family Pyrin Domain Containing 3 (NLRP3) (Wani et al., 2021; Taru et al., 2024). The activation of the nuclear factor-kappa B (NF-κB) and NLRP3 inflammasome pathways in adipose tissue and the liver promotes the release of pro-inflammatory cytokines (Sayaf et al., 2024). This inflammatory milieu disrupts insulin signaling and lipid metabolism, contributing to the progression of metabolic syndrome. Furthermore, macrophage infiltration into adipose tissue exacerbates inflammation and insulin resistance, creating a vicious cycle of metabolic dysfunction (Alemany, 2024).

### 4.4 Gut microbiota dysbiosis

The gut microbiota also plays a critical role in the development of metabolic syndrome through interactions with the host metabolism and immune system (Afzaal et al., 2022). Dysbiosis, or an imbalance in gut microbial composition, can promote metabolic endotoxemia by increasing the translocation of lipopolysaccharides (LPS) into the bloodstream (Charitos et al., 2025; Murugesan et al., 2025). LPS triggers systemic inflammation and disrupts insulin sensitivity. In contrast, beneficial gut bacteria produce short-chain fatty acids (SCFAs) such as butyrate, which exert anti-inflammatory effects and enhance gut barrier function (Yu et al., 2025). Dietary interventions that promote gut microbial diversity and SCFA production have shown promise in alleviating metabolic syndrome (Donati Zeppa et al., 2024; Facchin et al., 2024).

### 4.5 Lipid dysregulation

Another critical aspect of metabolic syndrome is lipid dysregulation, which manifests as elevated triglycerides, low high-density lipoprotein (HDL) cholesterol, and increased low-density lipoprotein (LDL) cholesterol (Alcover et al., 2025). This dyslipidemia is partly due to hepatic insulin resistance, which impairs the suppression of hepatic glucose production and enhances very low-density lipoprotein (VLDL) secretion (Jahdkaran and Sistanizad, 2025). Additionally, increased FFA flux to the liver promotes triglyceride synthesis and steatosis, further contributing to metabolic disturbances (Folestad and Falkevall, 2024).

### 4.6 Hypertension and vascular dysfunction

Insulin resistance, endothelial dysfunction, and an overactive renin-angiotensin system (RAS) all contribute to hypertension in the metabolic syndrome (Zamolodchikova et al., 2024). When cells become insulin resistant, the pancreas overproduces insulin (hyperinsulinemia), resulting in kidney salt retention, increased vascular smooth muscle growth, and sympathetic nervous system overstimulation, all of which raise blood pressure (Brosolo et al., 2022). At the same time, damaged blood vessel linings (endothelial dysfunction) reduce nitric oxide (NO) production, limiting blood vessel relaxation and increasing vascular resistance. Furthermore, imbalanced RAS leads to blood vessel constriction and increased salt retention (Gallo et al., 2022). Together, these systems create a vicious cycle that keeps blood pressure high and aggravates metabolic syndrome (Kurhaluk and Tkaczenko, 2025).

### 4.7 Genetic and epigenetic factors

Genetic and epigenetic factors also influence the susceptibility to metabolic syndrome. Polymorphisms in genes involved in glucose and lipid metabolism, insulin signaling, and inflammatory pathways can increase the risk of developing metabolic syndrome (Ercegović et al., 2025). Epigenetic modifications, such as DNA methylation and histone acetylation, may be influenced by environmental factors like diet and physical activity, further modulating metabolic outcomes (Ray et al., 2024).

## 5 Gut microbiota, inflammation and metabolic syndrome

**Figure 2:**
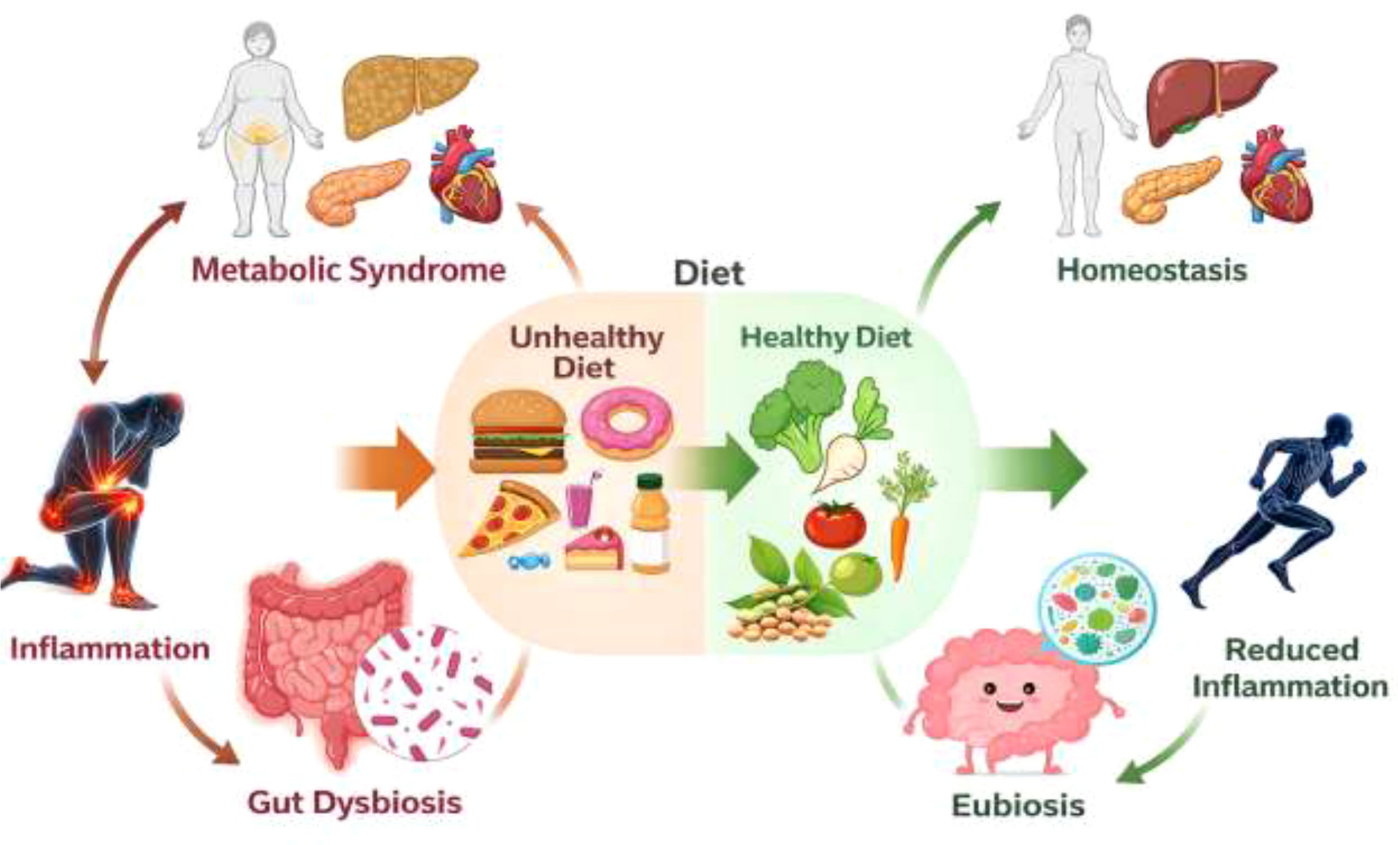
The interaction between gut microbiome, inflammation and metabolic syndrome.

The gut microbiota consists of numerous bacteria, viruses, fungi, and other microorganisms that play pivotal roles in regulating immune responses and inflammation (Chandrasekaran et al., 2024). This microbial community supports vital processes such as digestion, metabolism, and immune defense (Kumari et al., 2024). However, disturbances in gut microbiota composition, known as dysbiosis, can drive chronic low-grade inflammation, a hallmark of metabolic syndrome, which encompasses conditions like obesity, insulin resistance, dyslipidemia, and hypertension (Hou et al., 2022). Emerging evidence suggests that dysbiosis disrupts homeostasis through mechanisms such as impaired microbial metabolite production, immune signaling alterations, and compromised gut barrier integrity (**Figure 2**).

A balanced gut microbiota is characterized by diversity, symbiosis, and robust metabolic activity, with beneficial bacterial genera such as Bifidobacterium, Lactobacillus, and Akkermansia contributing to intestinal homeostasis (Kumar et al., 2025; Profir et al., 2025). Conversely, pathogenic species like *Escherichia coli* and *Clostridioides difficile* can promote inflammation when they proliferate (Lucas and Brown, 2024; Spigaglia, 2024; Gurung et al., 2025). Disruptions to microbial equilibrium are often caused by poor dietary habits, antibiotic overuse, chronic stress, and sedentary lifestyles. Diets high in saturated fats and low in dietary fiber reduce beneficial bacteria while fostering pro-inflammatory species, further exacerbating dysbiosis (Zhou et al., 2024).

One critical mechanism through which gut bacteria influence inflammation involves the production of short-chain fatty acids (SCFAs), such as butyrate, acetate, and propionate (Zhou et al., 2024). These metabolites, generated through dietary fiber fermentation, exert anti-inflammatory effects by modulating immune pathways. Butyrate suppresses nuclear factor-kappa B (NF-κB), a key regulator of inflammatory gene expression, and enhances gut barrier function by upregulating tight junction proteins like occludin and ZO-1 (Saban Güler et al., 2025). Studies, including those by Altemani et al (Altemani et al., 2021), have shown that reduced levels of butyrate-producing bacteria, such as Coprococcus, compromise intestinal barrier integrity, leading to metabolic endotoxemia, a condition where bacterial toxins like lipopolysaccharides (LPS) enter systemic circulation. This triggers pro-inflammatory cytokine release, exacerbating insulin resistance and other metabolic dysfunctions. SCFAs also activate G-protein-coupled receptors (GPR41 and GPR43) on immune cells, reducing pro-inflammatory cytokines like TNF-α and IL-6 while promoting regulatory T cell (Treg) differentiation, which maintains immune tolerance.

Conversely, harmful bacterial products such as LPS exacerbate inflammation and metabolic dysfunction. LPS, a component of Gram-negative bacteria, binds to Toll-like receptor 4 (TLR4), activating NF-κB and the NLRP3 inflammasome, leading to the release of IL-1β and TNF-α (Page et al., 2022). Chronic exposure to low-grade LPS, as seen in metabolic endotoxemia, drives insulin resistance and adipose tissue inflammation. High-fat diets worsen this by promoting Gram-negative bacterial overgrowth and increasing intestinal permeability (Chen et al., 2025). Additionally, gut barrier dysfunction is further aggravated by dysbiosis-induced reductions in mucus production and tight junction integrity. *Akkermansia muciniphila*, a mucin-degrading bacterium, plays a crucial role in maintaining the mucus layer, and its depletion is associated with increased gut permeability and systemic inflammation (Kumar et al., 2025; Profir et al., 2025).

Evidence linking gut dysbiosis to metabolic syndrome includes microbial composition alterations, such as an elevated Firmicutes-to-Bacteroidetes ratio in obese individuals, which enhances energy extraction and adiposity (Portincasa et al., 2022). Reduced SCFA-producing bacteria further exacerbate inflammation and metabolic dysfunction. Therapeutic strategies targeting the gut microbiota, such as dietary interventions with prebiotic fibers and polyphenol-rich foods, have shown promise in restoring microbial balance (Yarahmadi et al., 2024). Probiotic supplementation with Lactobacillus and Bifidobacterium strains can reduce inflammation, while fecal microbiota transplantation (FMT) may restore microbial diversity in severe dysbiosis (Farah et al., 2025). Studies by Sevillano-Jiménez et al. (2022) demonstrate that prebiotic and probiotic interventions improve metabolic parameters by enhancing gut barrier function and reducing permeability. Similarly, Liu et al. (2021) highlight the potential of fiber and probiotics in mitigating metabolic side effects by decreasing LPS-induced inflammation.

Lifestyle modifications, including regular physical activity and stress management, also play a significant role in maintaining gut health (Aziz et al., 2024). Exercise enhances microbial diversity and SCFA production, while mindfulness practices and adequate sleep reduce gut permeability and inflammation (Meher et al., 2024; Yao et al., 2025). Research by Chumpitazi et al. (2021) underscores how microbial imbalances, such as those seen in irritable bowel syndrome (IBS), contribute to chronic inflammation and metabolic disturbances. Furthermore, studies on non-alcoholic steatohepatitis (NASH) by De Oliveira et al. (2020) reveal that pro-inflammatory bacterial species exacerbate liver dysfunction and metabolic complications. Collectively, these findings emphasize the importance of preserving gut microbial homeostasis through dietary, probiotic, and lifestyle interventions to prevent and manage metabolic syndrome (**Table 1**). By addressing dysbiosis and its inflammatory consequences, these strategies offer a promising approach to mitigating the growing burden of metabolic disorders.

**Table 1:** Studies showing the mechanism linking gut microbiota and inflammation in metabolic syndrome.

Study	Aim of the study	Key insights/findings	Mechanism linking gut microbiota and inflammation in metabolic syndrome
Intermittent Fasting Improves Cardiometabolic Risk Factors and Alters Gut Microbiota in Metabolic Syndrome Patients (Guo et al., 2021)	To investigate the impact of intermittent fasting on cardiometabolic risk factors and gut microbiota composition in patients with metabolic syndrome.	Intermittent fasting improves insulin sensitivity, reduces inflammation, and modifies gut microbiota composition.	Fasting alters gut microbiota diversity, reducing pro-inflammatory bacteria and improving metabolic endotoxemia.
Improvement of Lipoprotein Profile and Metabolic Endotoxemia by a Lifestyle Intervention That Modifies the Gut Microbiota in Subjects With Metabolic Syndrome (Guevara‐Cruz et al., 2019)	To evaluate how lifestyle interventions affect lipoprotein profiles and metabolic endotoxemia via gut microbiota modulation.	Lifestyle changes improve lipoprotein profiles and reduce metabolic endotoxemia through gut microbiota modification.	Enhanced gut microbiota diversity reduces endotoxin-producing bacteria, decreasing systemic inflammation.
Supplementation with *Akkermansia muciniphila* in Overweight and Obese Human Volunteers: A Proof-of-Concept Exploratory Study (Depommier et al., 2019)	To assess the effects of *Akkermansia muciniphila* supplementation on metabolic health and inflammation in overweight individuals.	Supplementation with *Akkermansia muciniphila* improves insulin sensitivity and reduces inflammation.	*Akkermansia muciniphila* restores gut barrier integrity, reducing endotoxin leakage and systemic inflammation.
Effect on Gut Microbiota of a 1-y Lifestyle Intervention with Mediterranean Diet Compared with Energy-Reduced Mediterranean Diet and Physical Activity Promotion: PREDIMED-Plus Study (Muralidharan et al., 2021)	To compare the effects of two Mediterranean diet approaches on gut microbiota and metabolic health over a year.	Mediterranean diet alters gut microbiota, reducing pro-inflammatory markers and improving metabolic health.	Increased short-chain fatty acid (SCFA) production reduces inflammation and improves metabolic regulation.
Effects of Synbiotic Supplementation on Metabolic Syndrome Traits and Gut Microbial Profile among Overweight and Obese Hong Kong (Lauw et al., 2023) Chinese Individuals: A Randomized Trial	To evaluate the effects of synbiotic supplementation on metabolic syndrome traits and gut microbiota in overweight Chinese individuals.	Synbiotic supplementation reduces BMI, improves lipid profile, and modulates gut microbiota composition.	Synbiotics enhance beneficial bacteria, reducing inflammation via modulation of gut barrier and immune response.
Exercise Training Modulates the Gut Microbiota Profile and Impairs Inflammatory Signaling Pathways in Obese Children (Quiroga et al., 2020)	To examine how exercise training influences gut microbiota and inflammatory pathways in obese children.	Exercise training reduces inflammation and modulates gut microbiota composition in obese children.	Exercise-induced microbiota changes reduce pro-inflammatory cytokines and improve gut permeability.
Dietary Modulation of Gut Microbiota Contributes to Alleviation of Both Genetic and Simple Obesity in Children (Zhang et al., 2015)	To investigate how dietary changes impact gut microbiota and alleviate obesity-related inflammation in children.	Dietary intervention reduces obesity-related inflammation and modulates gut microbiota.	Increased fiber intake enhances SCFA production, reducing systemic inflammation and metabolic dysfunction.
A Purified Membrane Protein from *Akkermansia muciniphila* or the Pasteurized Bacterium Improves Metabolism in Obese and Diabetic Mice (Plovier et al., 2017)	To explore the metabolic benefits of a purified membrane protein from *Akkermansia muciniphila* in obese and diabetic mice.	Pasteurized *Akkermansia muciniphila* improves glucose metabolism and reduces inflammation.	Enhanced gut barrier integrity reduces endotoxin translocation and systemic inflammation.
Metabolome-associated psychological comorbidities improvement in irritable bowel syndrome patients receiving a probiotic (Martin et al., 2024)	Modulation of gut microbiota influences systemic metabolite production, which affects inflammation and mental health in metabolic disorders.	To evaluate the effects of probiotics on metabolome-associated psychological symptoms in IBS patients.	Probiotic supplementation improved psychological symptoms and altered gut metabolite profiles.
Probiotic or Synbiotic Alters the Gut Microbiota and Metabolism in a Randomised Controlled Trial of Weight Management in Overweight Adults (Hibberd et al., 2019)	To assess how probiotics and synbiotics influence gut microbiota and metabolic outcomes in overweight adults.	Probiotics and synbiotics improve metabolic parameters and gut microbiota composition.	Probiotics reduce inflammation by increasing anti-inflammatory bacterial strains and reducing endotoxins.
Effects of fecal microbiota transplant on DNA methylation in subjects with metabolic syndrome (Van Der Vossen et al., 2021)	Alterations in gut microbiota affect host epigenetics, contributing to inflammation and metabolic dysfunction.	To assess the impact of fecal microbiota transplantation (FMT) on DNA methylation patterns in metabolic syndrome patients.	FMT induced changes in DNA methylation related to metabolic pathways and inflammation.
Red wine polyphenols modulate fecal microbiota and reduce markers of the metabolic syndrome in obese patients (Moreno-Indias et al., 2016)	Moreno-Indias I et al., 2016. DOI: 10.1039/c5fo00886g	To investigate the effects of red wine polyphenols on gut microbiota and metabolic syndrome markers.	Red wine polyphenols improved gut microbiota composition and reduced inflammation and metabolic markers.
Lactobacillus casei Shirota Supplementation Does Not Restore Gut Microbiota Composition and Gut Barrier in Metabolic Syndrome (Stadlbauer et al., 2015)	Limited probiotic effects suggest that gut microbiota dysbiosis in metabolic syndrome is resilient to simple probiotic supplementation.	To examine whether Lactobacillus casei Shirota restores gut microbiota composition and gut barrier function.	The probiotic did not significantly restore gut microbiota or improve gut barrier integrity in metabolic syndrome.
Effects of Metabolic Syndrome on Intestinal Flora, Inflammatory Factors, and Infants of Pregnant Patients (Zong-Jie and Zhen, 2020)	Metabolic syndrome disrupts gut microbiota, promoting pro-inflammatory cytokines that impact maternal and fetal health.	To explore how metabolic syndrome affects gut microbiota, inflammation, and pregnancy outcomes.	Metabolic syndrome altered maternal gut flora and increased inflammatory markers, affecting infant health.
Probiotic and synbiotic supplementation could improve metabolic syndrome in prediabetic adults (Kassaian et al., 2019)	Probiotics and synbiotics modulate gut flora to decrease inflammatory markers and improve metabolic health.	To investigate the effects of probiotics on metabolic syndrome parameters in prediabetic adults.	Probiotic and synbiotic supplementation improved lipid profiles and reduced inflammation.
Contrasting effects of fresh and fermented kimchi consumption on gut microbiota composition and gene expression related to metabolic syndrome (Han et al., 2015)	Fermented foods enhance beneficial gut bacteria that regulate inflammation and metabolic pathways.	To compare how fresh vs. fermented kimchi affects gut microbiota and metabolic gene expression.	Fermented kimchi improved gut microbiota diversity and metabolic gene regulation more effectively than fresh kimchi.
Prognostic value of a combination of innovative factors (gut microbiota, sarcopenia, obesity, metabolic syndrome) to predict surgical/oncologic outcomes following surgery for sporadic colorectal cancer (Veziant et al., 2020)	Dysbiosis and metabolic syndrome drive inflammation, impacting cancer prognosis.	To investigate how gut microbiota and metabolic syndrome predict colorectal cancer outcomes.	Gut microbiota composition and metabolic syndrome markers predicted post-surgical outcomes.
Fecal Microbial Transplantation and Fiber Supplementation in Patients with Severe Obesity and Metabolic Syndrome (Mocanu et al., 2021)	To evaluate the combined effect of fecal microbiota transplantation and fiber supplementation on metabolic outcomes in severely obese patients.	Fecal microbiota transplantation combined with fiber reduces inflammation and improves metabolic outcomes.	Transplanted gut microbiota increases SCFA production, improving gut integrity and reducing inflammation.
Non-alcoholic steatohepatitis: Comparison of intestinal microbiota between different metabolic profiles (De Oliveira et al., 2020)	Dysbiotic microbiota produces pro-inflammatory metabolites that activate immune pathways, promoting inflammation and metabolic dysfunction.	To compare gut microbiota composition in patients with different metabolic profiles suffering from non-alcoholic steatohepatitis (NASH).	NASH patients showed gut microbiota dysbiosis characterized by increased pro-inflammatory bacterial species.
Pregnant women who develop preeclampsia have lower abundance of *Coprococcus* in their gut microbiota (Altemani et al., 2021).	Reduced butyrate levels from decreased *Coprococcus* abundance may impair gut barrier integrity, leading to systemic inflammation associated with metabolic syndrome.	To investigate the relationship between gut microbiota composition and preeclampsia in pregnant women.	Women with preeclampsia had a significantly lower abundance of *Coprococcus*, a butyrate-producing bacterium.
Prebiotic Treatment in Patients with Nonalcoholic Fatty Liver Disease (NAFLD)-A Randomized Pilot Trial (Reshef et al., 2024)	Prebiotics promote beneficial bacteria, reducing inflammation and improving metabolic parameters.	To evaluate prebiotics’ effects on gut microbiota and inflammation in NAFLD patients.	Prebiotic treatment improved gut microbiota composition and reduced markers of inflammation.
Fructan-sensitive children with irritable bowel syndrome have distinct gut microbiome signatures (Chumpitazi et al., 2021)	Altered gut microbiota composition in fructan-sensitive individuals triggers immune activation and inflammation, contributing to metabolic disturbances.	To identify differences in gut microbiome composition in fructan-sensitive children with irritable bowel syndrome (IBS).	Children with fructan sensitivity exhibit distinct microbial profiles, characterized by reduced diversity and increased pro-inflammatory taxa.
Dietary fiber and probiotics for the treatment of atypical antipsychotic-induced metabolic side effects (Liu et al., 2021).	Fiber and probiotics may restore gut microbiota balance, reduce lipopolysaccharide (LPS) production, and lower inflammation linked to metabolic syndrome.	To examine the effects of dietary fiber and probiotics on metabolic side effects induced by antipsychotic medications.	Study protocol outlines plans to assess whether dietary interventions mitigate weight gain and metabolic abnormalities.
Impact of high prebiotic and probiotic dietary education on cardio-metabolic profile in schizophrenia spectrum disorders (Sevillano-Jiménez et al., 2022).	Probiotics and prebiotics modulate gut microbiota, reducing gut permeability and systemic inflammation, which are key contributors to metabolic syndrome.	To evaluate how prebiotic and probiotic dietary education affects the cardio-metabolic profile of patients with schizophrenia during the COVID-19 era.	Prebiotic and probiotic dietary intervention improved metabolic parameters, including reduced inflammation and better lipid profiles.
Impact of oral vancomycin on gut microbiota, bile acid metabolism, and insulin sensitivity (Vrieze et al., 2014)	Antibiotics disrupt gut microbiota, impairing bile acid metabolism and promoting inflammation and insulin resistance.	To evaluate the effect of oral vancomycin on gut microbiota and metabolic parameters.	Vancomycin altered gut microbiota, decreased bile acid production, and worsened insulin resistance.

## 6 Food bioactives

Food bioactives are naturally occurring compounds found in plants and animal products that provide health benefits beyond basic nutrition (Sultana et al., 2025). Unlike macronutrients (carbohydrates, proteins, and fats) or micronutrients (vitamins and minerals), bioactives are not essential for survival but play a crucial role in preventing chronic diseases and promoting longevity (Kussmann et al., 2023). These compounds exhibit diverse biological activities, including antioxidant, anti-inflammatory, and antimicrobial effects, making them valuable in modern preventive medicine (Ahmed et al., 2021; Effiong et al., 2025b).

**Figure 3:**
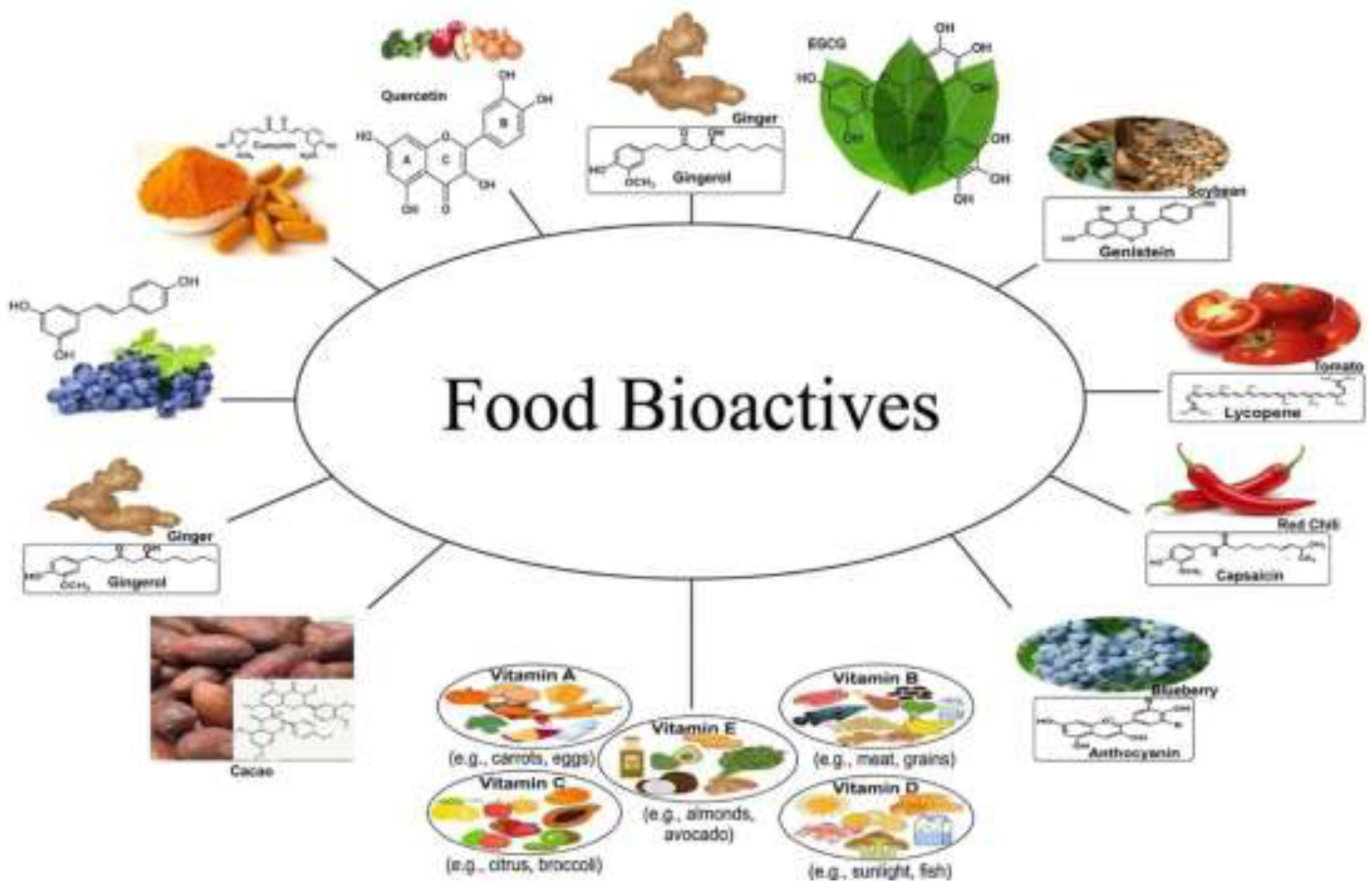
Various sources of food bioactives.

They consist of a diverse array of whole foods, each contributing unique health benefits. Polyphenols, one of the most studied groups of bioactives, are widely distributed in plant-based foods such as berries, green tea, dark chocolate, and spices like turmeric (**Figure 3**) (Effiong et al., 2024a; Effiong et al., 2025a). These compounds are responsible for the vibrant colors and distinct flavors of many fruits and vegetables while also providing potent antioxidant and anti-inflammatory effects (Mafe et al., 2025). Research has shown that the polyphenols in green tea, particularly catechins, may support cardiovascular health, while those in berries, such as anthocyanins, have been linked to improved cognitive function (Radeva-Ilieva et al., 2025).

Flavonoids, a subclass of polyphenols, are abundant in citrus fruits, onions, apples, and red wine. These compounds play a crucial role in modulating cellular signaling pathways, reducing inflammation, and enhancing immune function (Effiong et al., 2024b; Effiong et al., 2025a). Quercetin, a flavonoid found in apples and onions, has been studied for its potential to mitigate allergic responses and support respiratory health. The presence of these bioactives in commonly consumed foods makes them an accessible means of promoting long-term wellness (Alharbi et al., 2025).

Carotenoids, another important group of bioactives, are pigments that give fruits and vegetables their bright red, orange, and yellow hues (Roy et al., 2023). Found in carrots, tomatoes, spinach, and sweet potatoes, carotenoids such as beta-carotene and lycopene are known for their role in immune health. Beta-carotene, a precursor to vitamin A, supports vision and immune function, while lycopene, abundant in tomatoes, has been associated with a reduced risk of diseases (Tufail et al., 2024). These fat-soluble compounds are best absorbed when consumed with dietary fats, highlighting the importance of balanced meal preparation.

Omega-3 fatty acids, though distinct from plant-derived polyphenols and carotenoids, are equally vital bioactive compounds. They are primarily found in fatty fish like salmon and mackerel, as well as plant sources such as flaxseeds and walnuts (Swetha and Mathanghi, 2024; Tomas et al., 2025). These essential fatty acids, particularly eicosapentaenoic acid (EPA) and docosahexaenoic acid (DHA), are critical for brain health, reducing inflammation, and lowering the risk of cardiovascular diseases (Tomas et al., 2025). Studies suggest that regular consumption of omega-3-rich foods can improve lipid profiles and support cognitive function, making them a key component of a heart-healthy diet (Patted et al., 2024).

Glucosinolates, sulfur-containing compounds predominantly found in cruciferous vegetables like broccoli, Brussels sprouts, and kale, are another group of bioactives with significant health implications (Baldelli et al., 2025). When these vegetables are chopped or chewed, glucosinolates are broken down into bioactive metabolites such as sulforaphane, which has demonstrated anticancer properties in preclinical studies (Doodmani et al., 2025). These compounds are also known to support detoxification pathways in the liver, further underscoring their role in disease prevention.

### 6.1 Mechanism of action of food bioactives on gut microbiota and inflammation

Food bioactives such as polyphenols, flavonoids, and other bioactives demonstrate potential in ameliorating metabolic syndrome through multifaceted mechanisms (Afolabi et al., 2022; Adurosakin et al., 2023). These mechanisms encompass modulation of gut microbiota, reduction of inflammation, and improvement of metabolic parameters (**Table 2**). Polyphenols from camu camu (*Myrciaria dubia*) influence gut microbial composition, decrease inflammation, and increase energy expenditure (Abot et al., 2022; Agrinier et al., 2024). In diet-induced obese mice, camu camu treatment led to significant improvements in metabolic health via gut microbiota modulation.

**Table 2:** Mechanism of food bioactives/phytochemicals in regulating gut health, inflammation and metabolic syndrome.

Reference	Aim of study	Key insights/findings	Mechanism of food bioactives/phytochemicals
Naimi et al. (2021),	To investigate the direct effects of dietary emulsifiers on human gut microbiota composition.	Emulsifiers alter gut microbiota composition, increasing pro-inflammatory potential and disrupting gut barrier integrity.	Emulsifiers increase gut permeability, leading to the translocation of microbial products and subsequent inflammation.
Benameur et al. (2023),	To explore how bioactive compounds interact with gut microbiota to manage inflammatory diseases.	Bioactive compounds like polyphenols and flavonoids modulate gut microbiota, reducing inflammation.	Bioactives enhance beneficial microbial populations, decrease pro-inflammatory bacteria, and suppress cytokine release.
Peyrol et al. (2017),	To evaluate the protective effects of hydroxytyrosol against metabolic syndrome.	Hydroxytyrosol improves lipid metabolism, reduces oxidative stress, and mitigates inflammation.	Antioxidant properties of hydroxytyrosol reduce oxidative stress and inflammatory signaling.
Bertoncini‐Silva et al. (2023),	To assess the anti-obesity effects of bioactive dietary components.	Bioactive components improve energy metabolism and decrease adipose tissue inflammation.	Bioactives modulate metabolic pathways, reduce fat accumulation, and suppress inflammatory cytokines.
De Alvarenga et al. (2023),	To summarize the metabolic and health effects of monoterpenes.	Monoterpenes exhibit anti-inflammatory, antioxidant, and metabolic regulatory effects.	Modulate immune signaling, reduce oxidative stress, and improve lipid metabolism.
Fontes et al. (2019),	To evaluate the effects of mushroom-enriched diets on mitochondrial health in liver disease.	Mushroom bioactives protect mitochondria by reducing oxidative stress and promoting apoptosis in diseased cells.	Antioxidant bioactives reduce inflammation and maintain mitochondrial function.
Castelo et al. (2024),	To investigate the anti-inflammatory potential of phloroglucinol from gut microbiota.	Phloroglucinol reduces systemic inflammation and modulates the immune response.	Modulates gut microbiota to increase beneficial metabolites and reduce inflammation.
Chassaing et al. (2017),	To assess how dietary emulsifiers impact microbiota and inflammation.	Emulsifiers disrupt microbial balance and promote pro-inflammatory gene expression.	Alter gut microbiota and increase intestinal permeability, promoting inflammation.
Poulios et al. (2024),	To review the molecular mechanisms by which phytochemicals combat obesity.	Phytochemicals regulate lipid metabolism and suppress inflammation in adipose tissue.	Modulate metabolic enzymes, reduce inflammation, and enhance energy expenditure.
Liu et al. (2017),	To examine the effects of polymannuronic acid on obesity and inflammation.	Polymannuronic acid reduces obesity and inflammation through gut microbiota modulation.	Increases beneficial bacteria and reduces pro-inflammatory microbial metabolites.
Santa et al. (2023),	To explore the role of phytochemicals and Vitamin D in preventing metabolic syndrome.	Phytochemicals and Vitamin D can mitigate metabolic syndrome through anti-inflammatory and gut-modulating effects.	Polyphenols and Vitamin D improve gut microbiota composition, reduce inflammation, and regulate metabolic pathways.
Xiao et al (Uberti et al., 2017),	To review the evolution of phytochemicals from antioxidants to their role in gut health and metabolic syndrome.	Phytochemicals exhibit potent anti-inflammatory properties and modulate gut microbiota, improving metabolic outcomes.	Phytochemicals alter gut microbiota composition, reduce oxidative stress, and suppress pro-inflammatory pathways.
Santa et al. (2023),	To investigate how phytochemicals and Vitamin D contribute to disease prevention.	Synergistic effects of phytochemicals and Vitamin D improve immune responses and reduce chronic inflammation.	Vitamin D and phytochemicals modulate inflammatory cytokines and promote beneficial gut bacteria.
Komarnytsky et al. (2023),	To evaluate the effects of berries on microbiome-related metabolic and immune health.	Berry-derived polyphenols enhance gut microbiota diversity and reduce systemic inflammation.	Polyphenols in berries modulate gut microbial composition, improve gut barrier integrity, and suppress inflammatory markers.
Santa (2022),	To assess the role of grape phytochemicals and Vitamin D in lung disorder prevention.	Grape polyphenols and Vitamin D reduce inflammation and oxidative stress in lung disorders.	Polyphenols and Vitamin D suppress pro-inflammatory cytokines and promote antioxidant defense.
Liu et al. (2025),	To review the phytochemical composition and its effects on metabolic syndrome.	*Cynomorium songaricum* exhibits anti-inflammatory and metabolic regulatory effects.	Active compounds modulate gut microbiota, reduce inflammation, and improve insulin sensitivity.
Wang et al. (2024),	To elucidate the mechanisms by which rutin mitigates metabolic dysfunction in MAFLD.	Rutin improves lipid metabolism, reduces inflammation, and enhances gut microbiota.	Rutin modulates gut microbiota composition, suppresses pro-inflammatory mediators, and regulates lipid metabolism.
Anhê et al. (2019),	To investigate how camu camu affects obesity through gut microbiota modulation.	Camu camu alters gut microbiota, reduces inflammation, and increases energy expenditure.	Polyphenols in camu camu modulate gut microbiota and decrease systemic inflammation.
Feldman et al. (2021),	To assess the clinical efficacy of polyphenols in managing dyslipidemia.	Polyphenols significantly improve lipid profiles and reduce inflammation.	Polyphenols regulate lipid metabolism, modulate gut microbiota, and inhibit inflammatory pathways.
Zhang, Ji, et al (Zhou et al., 2024),	To analyze the impact of phytochemicals on ulcerative colitis using metabolomics and microbiome analysis.	Phytochemicals alleviate ulcerative colitis by modulating gut microbiota and metabolic pathways.	Phytochemicals restore gut microbiota balance, reduce inflammation, and improve intestinal health.
Poulios et al. (2024),	To highlight the molecular mechanisms of phytochemicals in obesity prevention.	Phytochemicals reduce adipogenesis, improve metabolism, and decrease inflammation.	Active compounds modulate lipid metabolism, alter gut microbiota, and suppress inflammatory markers.

**Figure 4:**
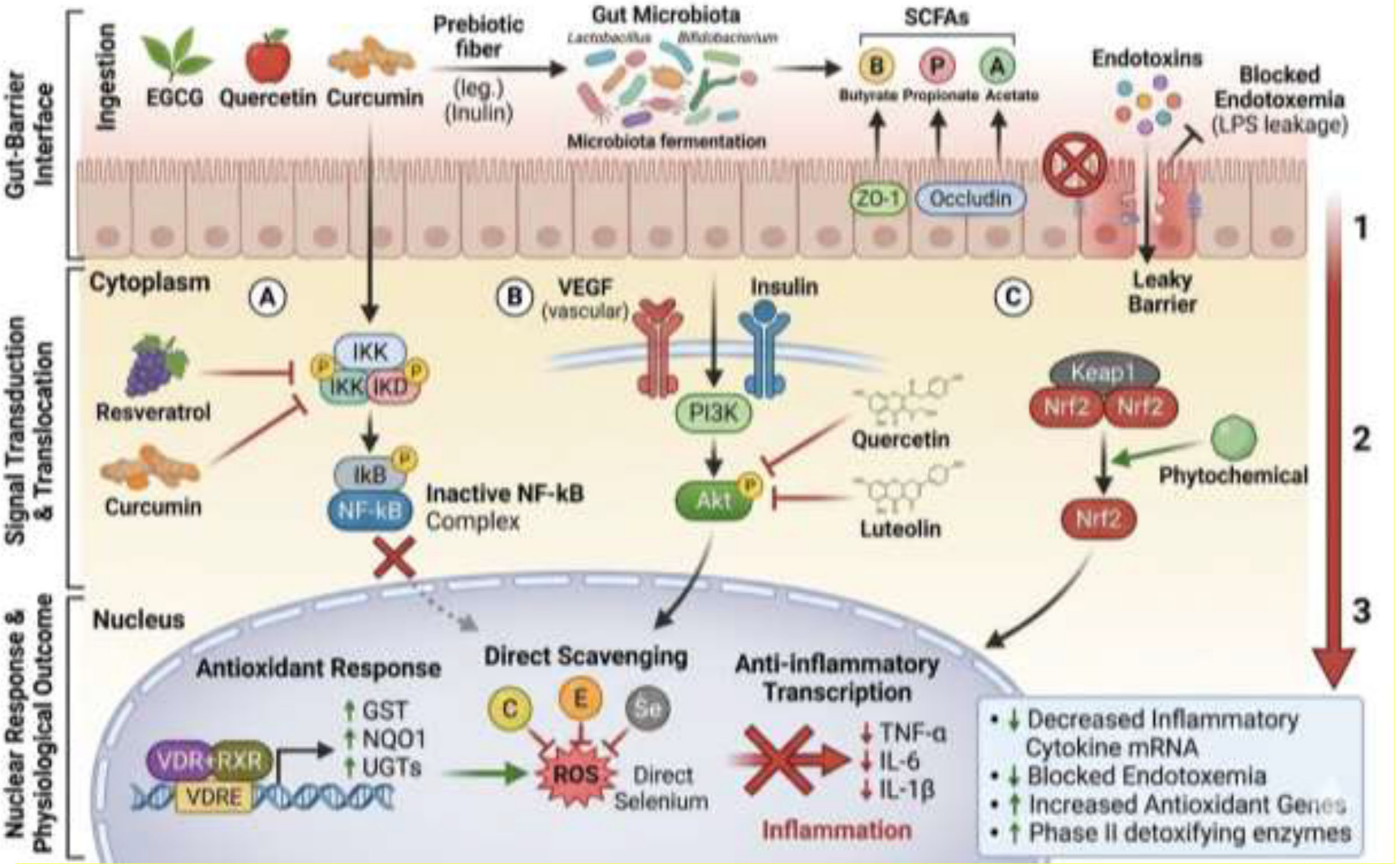
Mechanistic pathways of food bioactives on gut microbiome, inflammation and metabolic syndrome.

Grape polyphenols and Vitamin D exhibit synergistic effects in metabolic regulation (Quesada-Vázquez et al., 2024; Lopes et al., 2025). These bioactives modulate inflammatory cytokines and promote beneficial gut bacteria, collectively mitigating metabolic dysfunction (Santa et al., 2023). *Cynomorium songaricum*, a traditional medicinal plant, displays anti-inflammatory and metabolic regulatory properties by modulating gut microbiota and improving insulin sensitivity (Liu et al., 2025). Furthermore, phytochemicals restore gut microbiota balance and alleviate ulcerative colitis. Integrated metabolomics and microbiome analysis reveal that these bioactives reduce inflammation and improve intestinal health (Zhou et al., 2024). A study by Zhao et al. (2023) and Zhou et al (Zhou et al., 2024), found that quercetin and catechins modulate gut microbiota composition and reduce systemic inflammation, leading to improved metabolic profiles in obese individuals. Generally, bioactives from various dietary sources play a critical role in modulating gut microbiota, reducing inflammation, and improving metabolic outcomes. Through mechanisms involving anti-inflammatory pathways, antioxidant effects, and SCFA production, these compounds provide a therapeutic avenue for the management and prevention of metabolic syndrome and related inflammatory conditions (**Table 2**; **Figure 4**).

#### 6.1.1 Anti-inflammatory mechanisms through the NF-kB pathway

Nuclear Factor-κabba B family includes five transcription factors: RelA (p65), RelB, c-Rel, p100/p52 (NF-κB2), and p105/p50 (NFκB1) (Guo et al., 2024). The cytoplasm contains the latent NF-kB complex and IκB proteins, which need to be phosphorylated by the IκB kinase (IKK) complex. IKKc, a regulatory kinase, is responsible for activating NF-kB via both conventional and noncanonical pathways (Guo et al., 2024). Chronic inflammation in metabolic syndrome can activate NF-κB, resulting in various associate pathologies (Pavitra et al., 2023). Several natural phytochemicals have been shown to inhibit NF-κB activation, potentially reducing metabolic syndrome (Olayiwola and Gollahon, 2024). Curcumin, found in turmeric, disrupts the Inhibitory Kabba B Kinase (IKK) complex, reducing cell proliferation, and inflammation (Moon, 2024). Resveratrol, a grape-derived compound, inhibits the phosphorylation of IkBα, which normally precedes NF-κB nuclear translocation (Ma et al., 2015). Green tea’s EGCG, a major bioactive component, can suppress NF-κB by inhibiting IKK and promoting IkBα expression (Mokra et al., 2022). Rutin, a flavonoid abundantly found in citrus fruits and buckwheat, possesses anti-inflammatory properties by suppressing pro-inflammatory mediators and regulating lipid metabolism (Al-Dhabi et al., 2015), its dual action makes it particularly valuable in addressing the complex interplay between inflammation and metabolic disorders. Likewise, Zinc inhibits the activation of NFkB signalling, omega-3 fatty acids helps to reduce inflammation. They inhibit the formation of COX-2. They confer protection by acting through the G-Coupled Protein Receptors (GCPR) (Jarosz et al., 2017).

#### 6.1.2 Metabolic regulation through the PI3K/AkT signaling pathway

**Figure 5:**
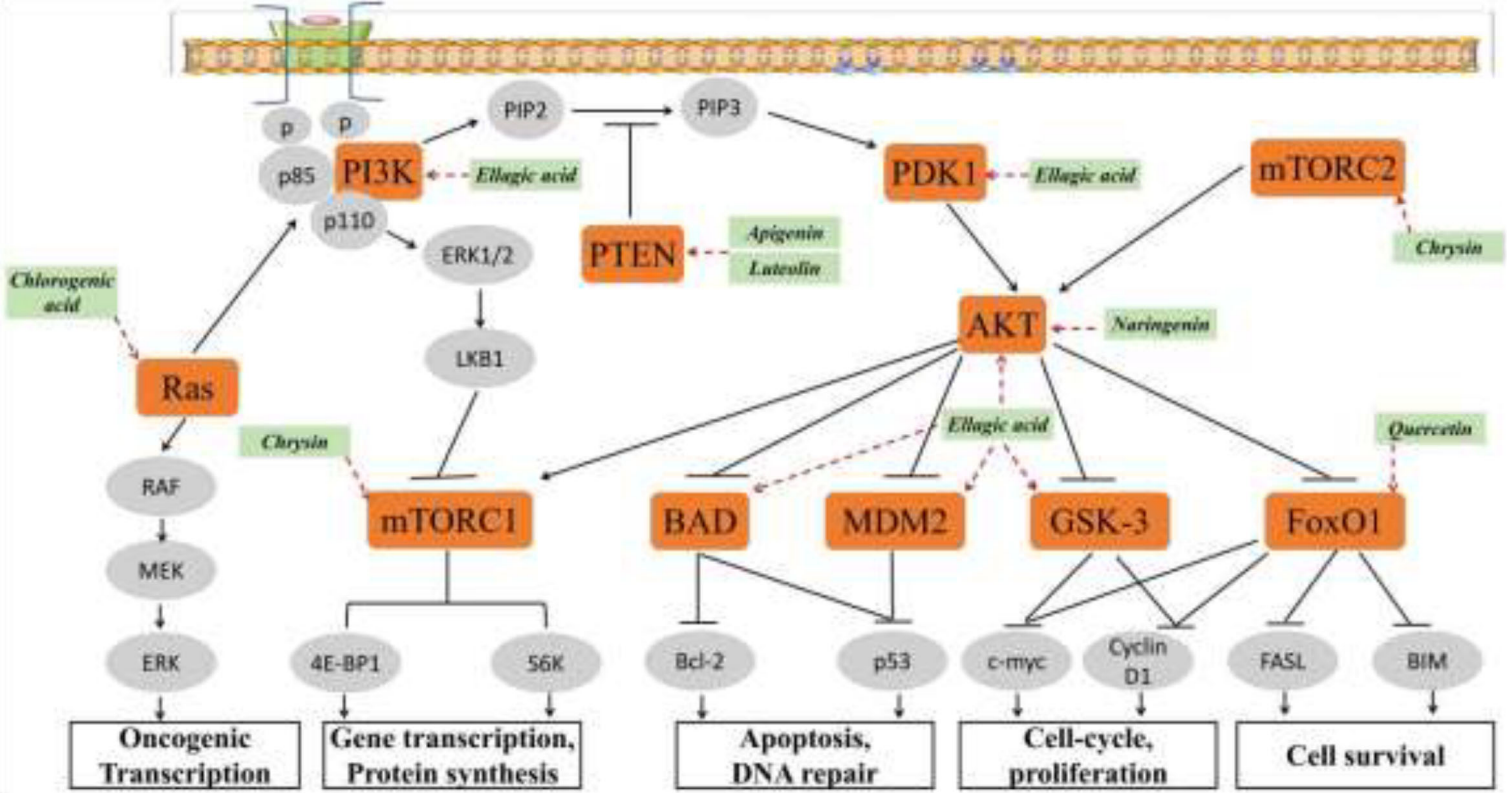
Action of bioactive compounds on PI3K/Akt signaling pathway (Effiong et al., 2025a).

The phosphoinositide 3-kinase (PI3K)/Akt signaling pathway plays a central role in metabolic regulation, inflammation, and host–microbiome interactions. Activation of the pathway occurs through growth factor receptors stimulated by ligands such as vascular endothelial growth factor (VEGF), insulin-like growth factor (IGF), platelet-derived growth factor (PDGF), epidermal growth factor (EGF), and fibroblast growth factor (FGF) (Effiong et al., 2026). This pathway plays a crucial role in metabolic syndrome, where it regulates insulin signaling, glucose uptake, lipid metabolism, and cellular inflammatory responses. Dysregulation of this pathway contributes to insulin resistance, chronic inflammation, and metabolic dysfunction, which are hallmark features of metabolic syndrome (Hopkins et al., 2020). Phytochemicals (**Figure 5**), such as curcumin, luteolin, resveratrol, and EGCG, have been shown to target this pathway (Effiong et al., 2025a). Curcumin, Apigenin, Quercetin inhibits PI3K activity and Akt phosphorylation (Aliyari et al., 2025), resveratrol, found in grapes and red wine, reduces cell migration and invasion, EGCG, the main bioactive component of green tea, downregulates Akt phosphorylation, inhibiting cell proliferation and inflammation (Mokra et al., 2022).

#### 6.1.3 Regulating gut microbiota, inflammation, and metabolic syndrome through vitamin D receptor signalling

The Vitamin D/VDR signaling pathway serves as a critical integrative axis for immune modulation, metabolic homeostasis, and gut microbiota composition. These biological effects are mediated through the Vitamin D Receptor (VDR), a ligand-activated nuclear transcription factor expressed in metabolically active tissues, including intestinal epithelial cells, adipose tissue, and pancreatic beta-cells (Carlberg and Campbell, 2013). Upon activation by the biologically active form of vitamin D, 1,25-dihydroxyvitamin D_3_ (calcitriol), the VDR forms a heterodimer with the Retinoid X Receptor (RXR). This complex translocates to the nucleus and binds to Vitamin D Response Elements (VDREs), initiating the transcription of genes essential for inflammation control, metabolic regulation, and barrier integrity (Carlberg and Campbell, 2013). In the context of metabolic syndrome, impaired Vitamin D signaling is a primary driver of insulin resistance and chronic low-grade inflammation. Mechanistically, VDR activation acts as a potent antagonist to NF-κB signaling, effectively suppressing the transcription of pro-inflammatory cytokines such as TNF-α, IL-6, and IL-1β. By attenuating this inflammatory cascade, Vitamin D signaling mitigates the systemic “metabolic noise” that contributes to cellular dysfunction. Furthermore, Vitamin D plays a foundational role in maintaining gut microbiota homeostasis and intestinal barrier integrity. VDR activation upregulates the production of antimicrobial peptides (e.g., cathelicidin and defensins) and enhances the expression of tight junction proteins, including occludin and claudins (Hamza et al., 2023). This strengthening of the physical barrier prevents the translocation of lipopolysaccharides (LPS) into systemic circulation, thereby reducing metabolic endotoxemia. Emerging evidence highlights a synergistic interplay where dietary bioactive compounds, such as polyphenols, flavonoids, and omega-3 fatty acids, enhance Vitamin D signaling. These compounds can modulate VDR expression and promote a microbial environment favorable to Vitamin D metabolism (Yao et al., 2024). Molecular mechanisms by which these phytochemicals might influence VDR signaling include modulating VDR expression and stability, enhancing co-activator recruitment, and crossing with other signaling pathways like PI3K/Akt, AMPK, and Nrf2 pathways (Cuenca-Micó and Aceves, 2020). Curcumin, derived from turmeric, enhances VDR expression and its interaction with co-activators (Goswami et al., 2022). Resveratrol, found in grapes and red wine, upregulates VDR expression (Uberti et al., 2017). Through cross-talk with the PI3K/Akt, AMPK, and Nrf2 pathways, Vitamin D signaling serves as a high-impact therapeutic target, where bioactive-driven sensitization of the VDR can lead to improved insulin sensitivity and a robust defense against oxidative stress.

#### 6.1.4 Antioxidant function of food bioactives

Natural bioactives, found in fruits, vegetables, and certain dietary sources, have been identified as potential anti-inflammatory agents due to their antioxidant properties (Effiong et al., 2024a; Effiong et al., 2025b). Phytochemicals with antioxidant properties act as scavengers, neutralizing free radicals and ROS before they damage cellular components (Nwozol and Effiong, 2019; Salemcity et al., 2020). Flavonoids like curcumin and quercetin directly scavenge free radicals and ROS, reducing oxidative stress and potentially preventing metabolic syndrome (Alharbi et al., 2025; Aliyari et al., 2025). Resveratrol, found in grapes and red wine, activates enzymes with antioxidant properties (Farhan and Rizvi, 2023). Vitamins and minerals also perform antioxidant functions, these include vitamin A, C, E and selenium mineral. Vitamin C is a water soluble antioxidant vitamin that plays key roles in scavenging free radicals, preventing their accumulation that leads to oxidative stress (Effiong et al., 2024c). In normal cells, vitamin C alternates between its oxidized form, Dihydroascorbic acid (DHA) and its reduced form, ascorbic acid in a reversible fashion. This enables it to accept and loss electrons from other free radicals that have the potential to cause damage to the macromolecules in the body (Effiong et al., 2024b).

#### 6.1.5 Oxidative stress regulation through the Nrf-2 signaling pathway

Oxidative stress, characterized by an imbalance between free radicals production and the antioxidant clearance capacity, leads to increased production of reactive oxygen species (ROS), which damage cellular components, contributes to inflammation and gut dysbiosis (Martemucci et al., 2023). The Nuclear factor erythroid 2–related factor 2 (Nrf-2) pathway plays a key role in oxidative stress regulation. Nrf-2 is a key transcription factor in the NrF-2 pathway that regulates cellular defense mechanisms against oxidative stress, inflammation, and metabolic dysfunction (He et al., 2020). It controls the expression of genes involved in antioxidant responses, detoxification, and cellular repair processes. In metabolic syndrome, chronic oxidative stress and low-grade inflammation disrupt metabolic homeostasis, contributing to insulin resistance, lipid dysregulation, and gut microbiome imbalance (Masenga et al., 2023). Activation of the Nrf-2 signaling pathway plays a protective role by enhancing antioxidant defenses and reducing inflammatory signaling, thereby mitigating metabolic disturbances associated with metabolic syndrome (Saha et al., 2020).

Food-derived bioactive compounds can activate the Nrf-2 pathway and promote metabolic health by inducing the expression of cytoprotective enzymes involved in detoxification and oxidative stress control (Thiruvengadam et al., 2021). These enzymes include glutathione-S-transferase (GST), NAD(P)H: quinone oxidoreductase 1 (NQO1), UDP-glycosyltransferases (UGTs), and catechol-O-methyltransferase (COMT), which facilitate the neutralization and elimination of reactive metabolites (Aranda-Rivera et al., 2022). Increased activity of these phase II detoxification enzymes enhances the body’s ability to manage oxidative stress and inflammatory mediators that contribute to metabolic syndrome (Ngo and Duennwald, 2022).

**Figure 6:**
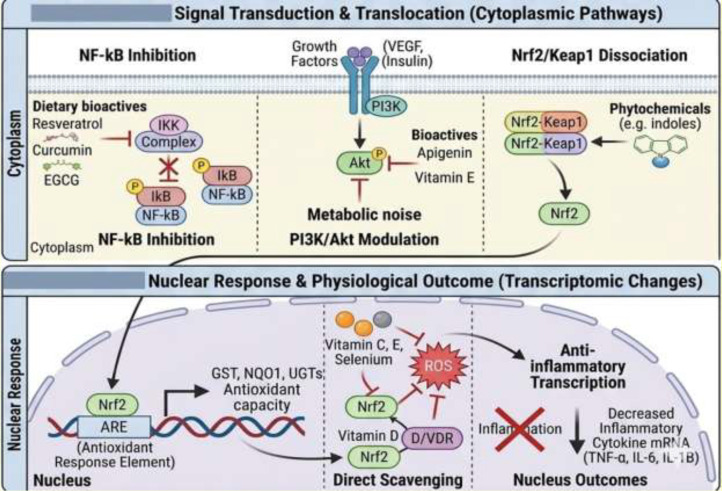
Action of food bioactives on cytoplasmic and nuclear signaling pathways.

Activation of Nrf-2 occurs primarily through two mechanisms: increased transcription of the Nrf-2 gene and enhanced release of Nrf-2 from its cytoplasmic inhibitor Kelch-like ECH-associated protein 1 (Keap1) (Ngo and Duennwald, 2022). Several dietary phytochemicals, including polyphenols, flavonoids, and indole-containing compounds, can stimulate Nrf-2 expression at the transcriptional level. These bioactives may also modulate epigenetic processes, such as DNA methylation, thereby enhancing Nrf-2 gene transcription and increasing cellular levels of the Nrf-2 protein. Once synthesized, Nrf-2 is normally sequestered in the cytoplasm by Keap1; however, phytochemicals can disrupt the Nrf-2–Keap1 interaction, allowing Nrf-2 to translocate into the nucleus. In the nucleus, Nrf-2 binds to antioxidant response elements (AREs), initiating the transcription of genes that encode antioxidant and detoxifying enzymes (Ngo and Duennwald, 2022) (**Figure 6**).

Beyond its antioxidant role, Nrf-2 activation also influences gut microbiome composition and intestinal barrier integrity. Bioactive compounds that stimulate Nrf-2 signaling can reduce oxidative stress within the intestinal environment, thereby supporting the growth of beneficial microbial communities and limiting the expansion of pathogenic bacteria associated with dysbiosis. Additionally, Nrf-2 activation improves gut barrier function and reduces endotoxin translocation, which in turn suppresses systemic inflammation commonly observed in metabolic syndrome (Thiruvengadam et al., 2021).

#### 6.1.6 Gut microbiome and immune regulation through short chain fatty acid production

**Figure 7:**
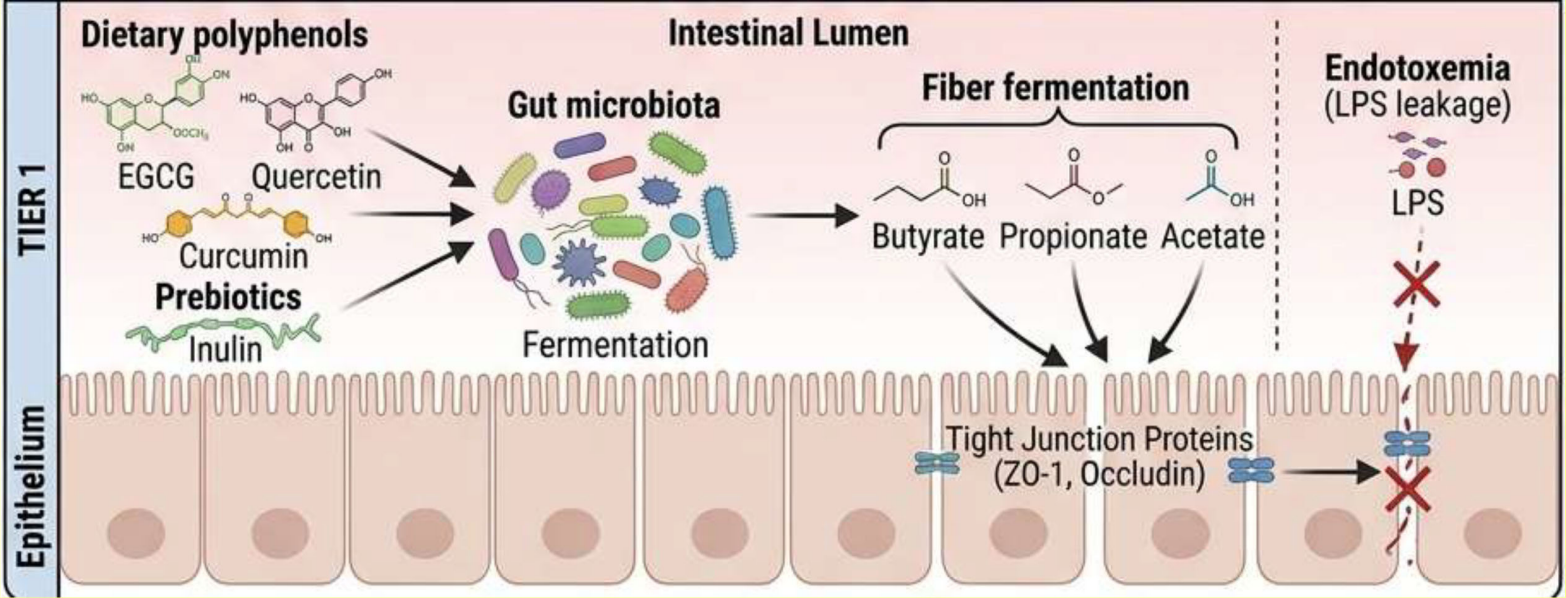
Effect of food bioactives on the gut microbiome.

Short-chain fatty acids (SCFAs), including butyrate, acetate, and propionate, represent crucial metabolic byproducts of dietary fiber fermentation by gut microbiota (Facchin et al., 2024). These microbial metabolites serve as fundamental mediators in the gut-immune axis, orchestrating a delicate balance between inflammatory responses and mucosal tolerance (**Figure 7**) (Zhou et al., 2025). At the molecular level, SCFAs exert their immunomodulatory effects primarily through interactions with G protein-coupled receptors (GPRs), particularly GPR43 and GPR109A (Yu et al., 2025). These receptors, abundantly expressed on intestinal epithelial cells and immune cells, serve as critical sensors for microbial metabolites (Lin and Zhang, 2017). When activated by SCFAs, GPR43 signaling initiates a cascade that suppresses nuclear factor-kappa B (NF-κB) activation, thereby reducing the production of pro-inflammatory cytokines such as tumor necrosis factor-alpha (TNF-α) and interleukin-6 (IL-6) (Chen et al., 2019). This receptor-mediated pathway represents a fundamental mechanism by which the gut microbiota communicates with the host immune system to maintain homeostasis.

Butyrate, the most extensively studied SCFA, demonstrates remarkable multi-target effects on gut barrier function. Through epigenetic modulation of tight junction proteins including occludin and zonula occludens-1 (ZO-1), butyrate significantly enhances intestinal epithelial integrity (Zhao et al., 2023). This barrier-strengthening effect is particularly crucial in preventing the translocation of pathogenic bacteria and their endotoxins, a phenomenon known as metabolic endotoxemia that underlies many chronic inflammatory conditions (Charitos et al., 2025). The ability of butyrate to maintain gut barrier competence explains its protective role against various intestinal disorders, including inflammatory bowel disease and metabolic syndrome.

The immunoregulatory capacity of SCFAs extends to their profound influence on immune cell differentiation and function. Butyrate’s inhibition of histone deacetylases (HDACs) creates an epigenetic landscape favorable for the development of regulatory T cells (Tregs) (Saban Güler et al., 2025). By increasing the expression of Foxp3, the master transcription factor for Treg development, butyrate promotes immune tolerance and prevents excessive inflammatory responses. This mechanism is particularly relevant in autoimmune conditions where Treg dysfunction contributes to disease pathogenesis (Singer et al., 2024). Furthermore, SCFAs have been shown to modulate dendritic cell function, promoting an anti-inflammatory phenotype that supports immune homeostasis.

The therapeutic potential of SCFAs has spurred interest in dietary interventions aimed at boosting their production. Prebiotic fibers such as inulin, found in foods like chicory root and Jerusalem artichokes, selectively nourish SCFA-producing bacteria including *Faecalibacterium prausnitzii* and *Roseburia* species (Kumar et al., 2025). Clinical studies demonstrate that inulin supplementation leads to measurable increases in fecal SCFA concentrations, accompanied by reductions in systemic inflammatory markers and improvements in insulin sensitivity (Abreu et al., 2022). These findings highlight the translational potential of SCFA-targeted nutritional strategies.

Beyond their local effects in the gut, SCFAs influence systemic immunity through circulation and direct interaction with peripheral immune cells. Acetate, the most abundant SCFA in circulation, has been shown to modulate B cell function and antibody production (Facchin et al., 2024). Propionate, meanwhile, influences neutrophil recruitment and function at distant sites of inflammation. This systemic immunomodulation underscores the far-reaching impact of gut-derived SCFAs on overall immune competence (Krsek and Baticic, 2024).

## 7 Limitation of the study

Despite the promising therapeutic potential of food bioactives in managing metabolic syndrome, several critical limitations impede their clinical application. The primary challenge lies in the low oral bioavailability and complex pharmacokinetics of compounds like curcumin and resveratrol, which often fail to reach effective systemic concentrations in human subjects. This is further compounded by significant inter-individual variability, or the “responder” phenomenon, where the host’s unique gut microbiota composition determines whether a bioactive is metabolized into its active form. Furthermore, much of the current mechanistic understanding is derived from animal models and isolated compounds, which may not accurately reflect the synergistic effects of a whole-food matrix or the long-term physiological complexities of human metabolic dysfunction. Standardizing dosages and accounting for diverse dietary patterns remain essential hurdles that future research must address through large-scale, longitudinal clinical trials.

## 8 Conclusion

The intricate interplay between the gut microbiota and systemic inflammation serves as a foundational driver in the pathogenesis of metabolic syndrome. This review has delineated how the disruption of gut barrier integrity leads to metabolic endotoxemia, triggering a cascade of pro-inflammatory signaling pathways—including NF-κB, PI3K/Akt, and Nrf2—that ultimately impair metabolic homeostasis. Evidence suggests that restoring microbial equilibrium through targeted dietary interventions, particularly the use of probiotics, prebiotics, and specific food bioactives, offers a robust therapeutic strategy. By promoting the production of short-chain fatty acids (SCFAs) and enhancing the expression of tight junction proteins, these interventions effectively mitigate chronic low-grade inflammation and improve insulin sensitivity. Future research should prioritize longitudinal clinical trials to explore the personalized therapeutic potential of microbiota modulation. Understanding inter-individual variability in response to bioactives will be crucial in developing precision nutrition frameworks for the effective prevention and management of metabolic syndrome and its associated cardiovascular complications.
